# Immunodeficiency-associated Vaccine-Derived Poliovirus Type 3 in Infant, South Africa, 2011

**DOI:** 10.3201/eid1806.120037

**Published:** 2012-06

**Authors:** Nicksy Gumede, Vongani Muthambi, Barry D. Schoub

**Affiliations:** National Institute for Communicable Diseases National Health Laboratory Service, Johannesburg, South Africa

**Keywords:** poliovirus, vaccine-derived poliovirus, oral polio vaccine, OPV, immunodeficiency, South Africa, viruses, polio, eradication, infant, vaccines, immunization, vaccination, IPV, inactivated poliovirus vaccine, *Suggested citation for this article*: Gumede N, Muthambi V, Schoub BD. Immunodeficiency-associated vaccine-derived poliovirus type 3 in infant, South Africa, 2011 [letter]. Emerg Infect Dis [serial on the internet]. 2012 June [*date cited*]. http://dx.doi.org/10.3201/eid1806.120037

## Abstract

Patients with primary immunodeficiency are prone to persistently excrete Sabin-like virus after administration of live-attenuated oral polio vaccine and have an increased risk for vaccine-derived paralytic polio. We report a case of type 3 immunodeficiency-associated vaccine-derived poliovirus in a child in South Africa who was born with X-linked immunodeficiency syndrome.

Live-attenuated oral polio vaccine (OPV) is still the vaccine of choice for use in developing countries. However, reversion to virulence may occur during OPV replication in humans and may result in the rare cases of vaccine-associated paralytic poliomyelitis in OPV recipients and their close contacts. Two additional OPV-related problems that may affect polio eradication: long-term, persistent infection with OPV-derived viruses in persons with primary humoral immunodeficiencies (so-called immunodeficiency-associated vaccine-derived polioviruses [iVDPVs]); and circulating vaccine-derived polioviruses (VDPV) in areas with low rates of vaccine coverage (*1*). VDPV strains are defined as follows: 1) strains of types 1 and 3, which have <99% nt sequence identity to the capsid viral protein (VP) 1 coding region of the corresponding Sabin reference strain; and 2) VDPV strains of type 2, which have <99.4% nt sequence identity to the corresponding Sabin reference viral protein 1 (VP1) (*1*). Circulating VDPVs show marked sequence drift, indicating prolonged replication of the vaccine strain in susceptible human hosts and consequent acquisition of the phenotypic properties of neurovirulence and transmissibility.

Persons born with primary immunodeficiency have been found to be persistently infected with VDPV after exposure to OPV. Immunocompetent persons excrete polio vaccine viruses for up to 2–3 months (*2*), whereas prolonged excretion of VDPV for 6 months to >10 years has been found in persons with primary humoral immunodeficiency (*3**–**6*). The risk for vaccine-associated paralytic poliomyelitis is >3,000-fold higher for these patients (*7*). We report a case of type 3 iVDPV in a child in South Africa who was born with X-linked immunodeficiency syndrome.

## The Patient

The patient, a 10-month-old boy, was born at term on October 28, 2010; X-linked immunodeficiency syndrome was diagnosed after he received 3 scheduled doses of polio vaccine (1 OPV dose at birth and 2 inactivated poliovirus vaccine doses at 10 and 14 weeks). On September 18, 2011, fever developed (38.5°C–40.0°C), and the next day, vomiting and 2 episodes of tonic-clonic convulsions occurred. A lumbar puncture was performed, and testing of cerebrospinal fluid (CSF) showed pleocytosis and mild increase of proteins. His condition deteriorated, and on day 5, acute flaccid paralysis developed, with generalized hypotonia and reduced power and reflexes in all limbs, more marked in the lower limbs. Respiratory distress developed, and some involvement of the facial nerve was manifested by left-sided eye drooping, mouth deviation, and drooling. A lumbar puncture was repeated on day 5, and CSF was positive by PCR for enterovirus and a pleocytosis. Stool samples taken on days 5 and 9 were positive for enterovirus, which was subsequently characterized as poliovirus type 3.

Beginning 15 days after the onset of paralysis, intravenous immunoglobulin (National Bioproducts Institute, KwaZulu-Natal, South Africa) with a titer for polio type 3 neutralizing antibodies of 4–8 IU was administered daily for 32 days, followed by alternate days to a total of 43 doses. The patient improved gradually, and strength was regained in all limbs, with the exception of residual paresis in the right lower limb. CSF became negative for poliovirus PCR 2 weeks after immunoglobulin therapy began, and stool excretion of poliovirus ceased on day 70, 55 days after initiation of immunoglobulin therapy.

Extracts of stool specimens were treated with chloroform and cultured on human rhabdomyosarcoma cell line, used for enterovirus isolation, and mouse L cells expressing the human poliovirus receptor, used specifically for poliovirus isolation (*8*). To distinguish whether the poliovirus isolates were of vaccine or wild origin, real-time PCR tests were performed, targeting the VP1 coding region (*9*). In addition, to detect mutant and recombinant poliovirus vaccine strains, a vaccine-derived, real-time screening assay was performed (David Kilpatrick, pers. comm.).

All Sabin 3 strains were sequenced at 3 regions of the genome: 5′ untranslated region, VP1, and 3D. The sequence analysis of all viruses revealed a mutation at nt 472 of the 5′ untranslated region (U_472_→C), a critical attenuating mutation feature for Sabin 3. This substitution in the internal ribosomal site restores the original structure of the stem loop and permitting the initiation of translation of the poliovirus RNA template (*10**,**11*) The reversion at that site is under strong selection during replication in the human intestine and is associated with the attenuated phenotype in Sabin 3 (*12*). The VP1 region showed 2 reversions of the capsid determinant; C_2493_→U appear to be the main determinants of the attenuated phenotype (*1*), and at position 54 for alanine amino acid mutated to valine (Ala_54_→Val) that can act as a suppressor of the temperature sensitivity and attenuated phenotype (*13*). At the 3D region, the sequence analysis showed no recombinant.

Both stool samples showed mixed bases at 12 positions, consistent with the presence of at least 2 main genetic variants in the virus population (Table). Isolates with mixed bases are characteristic of iVDPVs, which suggests the existence of co-replicating poliovirus lineages within immunodeficient patients (*1**,**5*).

**Table:** Mixed bases at various positions of immunodeficiency-associated vaccine-derived polioviruses

Sabin 3	Viral protein 1 position	Mixed bases	
		Stool sample 1	Stool sample 2
G	175	G/A	G/A
A	231	A/G	A/G
T	261	C/T	C/T
G	295	G/A	G/A
C	420	C/T	C
C	426	T/C	T/C
A	444	G/A	G/A
A	658	A/G	A/G
A	659	A/G	G/A
G	661	G/A	G/A
G	666	A/G	G/A
C	684	T/C	T/C

The relationships among the VP1 sequences of the 3 isolates were summarized in a tree constructed by using the neighbor-joining method (*14*) and rooted to the Sabin 3 sequence (Figure). The iVDVP isolates differed from the Sabin 3 OPV strain at 1.1% and from each other by 1.4% at a VP1 region, similar to the rate of nucleotide sequence evolution in poliovirus as described by Jorba et al. (*15*). The chronic iVDPV infection could have been initiated by the birth dose. The shallow branches correspond to 2 lineages (A, CSF, and B, stool). The extensive divergence of the two lineages was not surprising as the viruses originated from 2 sources (CSF and stool samples) taken 4 days apart. The VP1 sequence of lineage B was ambiguous at several positions, which suggests the virus population was of mixed variants. All sequences determined in this study were derived from Sabin 3 strain.

**Figure:**
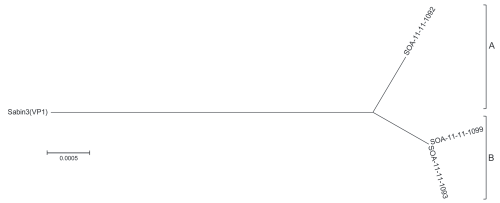
Neighbor-joining tree of immunodeficiency-associated vaccine-derived poliovirus isolates from infant, South Africa, 2011. The tree was derived from the viral protein (VP) 1 region and rooted at the Sabin 3 reference strain, showing classification as lineage A or B. South Africa (SOA) isolates are identified by 2 digits indicating year of receipt, 2 digits indicating year of onset of paralysis (*11*), and 4 digits indicating case number. SOA-11-11-1092 is a representative cerebrospinal fluid sample; SOA-11-11-1099 and SOA-11-11-1093 are representative stool samples. Scale bar indicates nucleotide substitutions per site.

## Conclusions

Cases of iVDPV are rare; especially rare is type 3. Only ≈50 cases had been reported in the literature as of March 2011 and, to our knowledge, none in sub-Saharan Africa. We characterized 2 separate lineages of type 3 poliovirus in this patient, demonstrating separate evolution of the virus. A relatively rapid clinical and virologic response to intravenous immunoglobulin averted chronic excretion of the virus. Persistent excretion of VDPV in primary immunodeficient patients remains a potential risk to the global eradication of polio, as long as OPV is still used.
